# Population genetics of four heavily exploited shark species around the Arabian Peninsula

**DOI:** 10.1002/ece3.1515

**Published:** 2015-05-20

**Authors:** Julia L Y Spaet, Rima W Jabado, Aaron C Henderson, Alec B M Moore, Michael L Berumen

**Affiliations:** 1 Red Sea Research Center, Division of Biological and Environmental Science and Engineering, King Abdullah University of Science and Technology23955-6900, Thuwal, Saudi Arabia; 2 Gulf Elasmo ProjectP.O. Box 29588, Dubai, United Arab Emirates; 3 Department of Marine Science & Fisheries, College of Agricultural & Marine Sciences, Sultan Qaboos UniversityMuscat, Oman; 4 RSK Environment Ltd, Spring LodgeHelsby, Cheshire, WA6 0AR, UK

**Keywords:** *Carcharhinus limbatus*, *Carcharhinus sorrah*, connectivity, elasmobranchs, *Rhizoprionodon acutus*, *Sphyrna lewini*

## Abstract

The northwestern Indian Ocean harbors a number of larger marine vertebrate taxa that warrant the investigation of genetic population structure given remarkable spatial heterogeneity in biological characteristics such as distribution, behavior, and morphology. Here, we investigate the genetic population structure of four commercially exploited shark species with different biological characteristics (*Carcharhinus limbatus*, *Carcharhinus sorrah*, *Rhizoprionodon acutus*, and *Sphyrna lewini*) between the Red Sea and all other water bodies surrounding the Arabian Peninsula. To assess intraspecific patterns of connectivity, we constructed statistical parsimony networks among haplotypes and estimated (1) population structure; and (2) time of most recent population expansion, based on mitochondrial control region DNA and a total of 20 microsatellites. Our analysis indicates that, even in smaller, less vagile shark species, there are no contemporary barriers to gene flow across the study region, while historical events, for example, Pleistocene glacial cycles, may have affected connectivity in *C. sorrah* and *R. acutus*. A parsimony network analysis provided evidence that Arabian *S. lewini* may represent a population segment that is distinct from other known stocks in the Indian Ocean, raising a new layer of conservation concern. Our results call for urgent regional cooperation to ensure the sustainable exploitation of sharks in the Arabian region.

## Introduction

Understanding the spatio-temporal patterns of gene flow among geographically separated populations has long been a major focus in ecology. Limited genetic differentiation over broad spatial scales is often associated with the high dispersal capacities of marine organisms, resulting from either a highly dispersive larval phase affected by ocean currents or the active movements of juvenile and adult specimens in animals lacking a planktonic larval stage. Yet, there are numerous well-known examples of barriers to gene flow within and among populations that result in higher than expected genetic structure, even in species with presumed high levels of vagility (e.g., dolphins: Andrews et al. 2010; Möller et al. 2011; killer whales: Foote et al. 2011; sharks: Blower et al. 2012; tuna: Dammannagoda et al. 2008; Kunal et al. 2013).

Patterns of genetic population structure in sharks are not uniform across species, but range from localized genetic subdivision (e.g., leopard shark: Lewallen et al. 2007; nurse shark: Karl et al. 2012; zebra shark: Dudgeon et al. 2009) and population structuring on relatively small geographic scales (e.g., blacktip reef shark: Vignaud et al. 2014a; bull shark: Karl et al. 2011; dusky shark: Benavides et al. 2011; grey nurse shark: Ahonen et al. 2009; lemon shark: Schultz et al. 2008; sandbar shark: Portnoy et al. 2010), to population differentiation detectable only across ocean basins (e.g., shortfin mako shark: Schrey and Heist 2003; whale shark: Castro et al. 2007; Schmidt et al. 2009; Vignaud et al. 2014b) and nearly global panmixia (basking shark: Hoelzel et al. 2006). Genetic subdivision in sharks is commonly facilitated by geographic dispersal barriers, such as large oceanic expanses (lemon shark: Schultz et al. 2008; spot-tail shark: Giles et al. 2014) or environmental gradients along continuous landmasses extending across different geographic regions (blacktip shark: Keeney and Heist 2006). In addition, the degree of species- and/or location-specific genetic differentiation is typically reflected by a combination of individual vagility, foraging habits, habitat preferences, reproductive mode, and sensitivity toward natural and anthropogenic influences (Dudgeon et al. 2012). The wide range of life histories and movement patterns exhibited by even closely related shark species hence hampers the a priori inference of spatial population structure.

**Figure 1:**
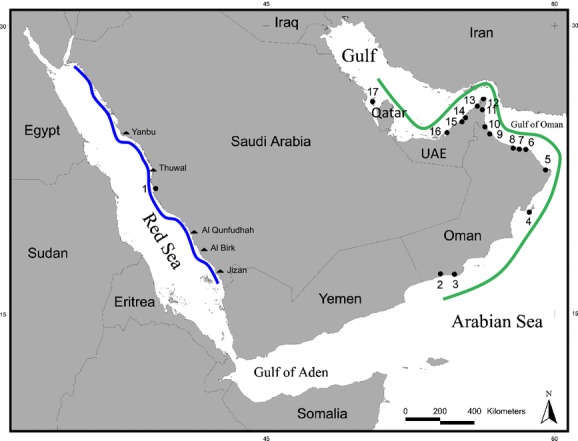
Map of the Arabian Sea region, displaying collection locations (circles) of *Carcharhinus limbatus*, *C. sorrah*, *Rhizoprionodon acutus,* and *Sphyrna lewini*. Numbers indicate fish markets or landing sites in Saudi Arabia, Oman, the United Arab Emirates, and Bahrain from where samples were obtained. (1) Jeddah, (2) Salalah, (3) Mirbat, (4) Masirah, (5) Sur, (6) Muscat, (7) Seeb, (8) Barka, (9) Sohar, (10) Shinas, (11) Dibba, (12) Khasab, (13) Ras Al Khaimah, (14) Sharjah, (15) Dubai, (16) Abu Dhabi, (17) Bahrain. See Table S1 for number of tissue samples obtained from each landing site or fish market. Triangles display other main landing sites in Saudi Arabia from which sharks are transported to the main fish market in Jeddah. Geographical color codes refer to haplotypes in Fig.2.

There is compelling evidence to investigate the genetic population structure of sharks in the water bodies surrounding the Arabian Peninsula, that is, the Arabian Sea, the Gulf of Oman and two semi-enclosed bodies of water, the Red Sea, and the Arabian/Persian Gulf (hereafter “the Gulf”) (Fig.1). First, a number of resident marine vertebrate taxa display remarkable heterogeneity in biological aspects, such as distribution, behavior, morphology, and population genetics. The Arabian Sea off the Oman coast, for instance, harbors the world's most isolated and most distinct population of nonmigratory humpback whales, *Megaptera novaeangliae* (Pomilla et al. 2014). Hawksbill turtles in the Gulf are significantly smaller than those in Omani waters (Pilcher et al. 2014), and sea snakes, which are abundant and diverse in the Gulf and present in the Arabian Sea, are entirely absent from the Red Sea (Sheppard et al. 1992). In addition, barriers to gene flow have been indicated between the Red Sea and the western Indian Ocean for several invertebrates (crabs: Fratini and Vannini 2002; sponges: Giles et al. in press) and some reef fishes (DiBattista et al. 2013), but not for others (Kochzius and Blohm 2005; DiBattista et al. 2013). In the Gulf, the large and highly mobile sailfish, *Istiophorus platypterus*, was described as phylogeographically isolated (Hoolihan et al. 2004), while another epipelagic predator, the Spanish mackerel, as well as the fiddler crab, does not appear to exhibit genetic subdivision between the Gulf and the Arabian Sea (Hoolihan et al. 2006; Shih et al. 2015).

Second, existing studies suggest variation in distributional and morphological patterns within Arabian elasmobranch species. Several elasmobranch species in the Arabian region have highly localized known distributions (e.g., *C. leiodon*: Moore et al. 2011) with a number of species endemic to the Red Sea (e.g., *H. bentuviai*: Baranes and Randall 1989) and the Gulf (e.g., *H. randalli*: Last et al. 2012). In addition, a large number of common elasmobranch species, which are reliably reported from the Gulf of Oman and the Gulf of Aden, have not been reported in the Gulf and the Red Sea, respectively (Moore 2011; Spaet et al. 2012). Furthermore, significant morphological differences between Gulf elasmobranchs and “typical forms” were suggested (Moore 2011), and a number of Gulf and Red Sea taxa still remain undescribed (unpublished data).

Recent global genetic studies of elasmobranchs have identified the Arabian region as one of four regions harboring a substantial proportion of taxa that are genetically distinct from their closest relatives in neighboring regions (Naylor et al. 2012). Moreover, global and range-wide studies on several species that included samples from ocean basins in the Arabian region demonstrated substantial genetic differentiation between this region and widely separated Indo-Pacific locations, as well as a strong separation between Indo-Pacific and Atlantic clades for blacktip reef (Vignaud et al. 2014a), silky (Clarke et al. 2015), spot-tail (Giles et al. 2014), and whale sharks (Schmidt et al. 2009; Vignaud et al. 2014b). Yet, in spite of the evident ecological distinctiveness of this region, no study to date has specifically focussed on the genetic population structure of elasmobranchs or indeed any other large vertebrate species around the Arabian Peninsula.

Despite its ecological relevance, the Arabian region features an alarming fisheries situation. Traditional and industrial shark fisheries exist throughout most of the region and for several countries have reached unsustainable exploitation levels (Bonfil 2003; Moore 2011; Jabado et al. 2014a; Spaet and Berumen 2015). Nonetheless, management strategies for shark resources are found in only a fraction of these countries, and proper enforcement of fisheries laws is essentially nonexistent (Bonfil 2003; Moore 2011; Spaet and Berumen 2015). In addition to an apparent general lack of concern toward the conservation of sharks in this region (Bonfil 2003; Spaet and Berumen 2015), the proper assessment and management of elasmobranch stocks has so far been hampered by insufficient information on the biology, ecology, and fisheries of exploited species (Moore 2011; Spaet et al. 2012). Only recently, efforts have been made to bridge this gap, contributing to our knowledge on country-specific fisheries and species-specific biological characteristics (Bonfil 2003; Henderson et al. 2006, 2007, 2009; Moore 2011; Spaet et al. 2011; Moore et al. 2012; Moore and Peirce 2013; Jabado et al. 2014a; Spaet and Berumen 2015).

Patterns of dispersal and population structure can vary significantly even among closely related species in shared habitats (Toonen et al. 2011; DiBattista et al. 2012). Therefore, in this study, we aimed to assess the genetic population structure of four shark species within the Arabian region with different biological, ecological, and life-history characteristics: (1) the blacktip shark, *Carcharhinus limbatus* (Müller & Henle, 1839); (2) the spot-tail shark, *Carcharhinus sorrah* (Müller & Henle, 1839); (3) the milk shark, *Rhizoprionodon acutus* (Rüppell, 1837); and (4) the scalloped hammerhead shark, *Sphyrna lewini* (Griffith & Smith, 1834).

*Carcharhinus limbatus* and *S. lewini* are found in coastal and semi-oceanic waters worldwide, although several studies suggest that undescribed diversity exists within both species (e.g., Zemlak et al. 2009; Naylor et al. 2012). *Carcharhinus sorrah* is found on continental and insular shelves, in the tropical and subtropical Indo-West Pacific, and *R. acutus* occurs along the continental shelf across the eastern Atlantic and Indo-West Pacific (Compagno 2001). *Rhizoprionodon acutus* is the smallest of the four species and reaches maximum total lengths (TL) of 98 cm in the study region while *C. sorrah*, *C. limbatus,* and *S. lewini* can reach 196 cm, 287 cm, and 303 cm TL, respectively (R. W. Jabado unpubl. data). Carcharhinids and Sphyrnids are placental livebearers with typically low intrinsic rates of increase. Although *S*.* lewini* exhibits the highest fecundity of all four study species, (12–41: White et al. 2008 cf. 1–11: Carrier et al. 2012 (range of the other three species)), resilience to exploitation is low due to the species' late age at maturity (10–30 years: Baum et al. 2007 cf. 2–7 years Compagno 1984). Based on International Union for Conservation of Nature (IUCN) Red List criteria, *R. acutus* is globally categorized as Least Concern, *C. limbatus* and *C. sorrah* are classified as Near Threatened, and *S. lewini* is listed as Endangered. Except for *R. acutus*, dispersal capacities for all species are considered very high. Tagging studies of *C. sorrah* demonstrated an individual maximum travel distance of 1116 km, although almost half of the tagged specimens were recaptured within 50 km of the tagging location (Stevens et al. 2000). Movements of up to 2148 km were observed for *C. limbatus* (Kohler et al. 1998), and an individual *S. lewini* specimen has reportedly traversed 1600 km of deep ocean habitat (Kohler and Turner 2001). Although no movement studies are available for *R. acutus*, the smaller body size of this species implies lower vagility compared to the three larger species, potentially indicating greater genetic subdivision.

We use a combination of mitochondrial (control region (CR)) and nuclear (microsatellites) markers. Congruence between both types of markers has been shown to yield a high degree of intraspecific resolution, providing a useful tool for the delineation of marine lineages and populations (e.g., Nance et al. 2011; Ovenden et al. 2011). Moreover, contrasting nuclear and mitochondrial data have been used successfully to identify sex-biased dispersal patterns in different elasmobranch species (e.g., Pardini et al. 2001; Portnoy et al. 2010; Daly-Engel et al. 2012). By combining two kinds of genetic markers over four species with variable biology, life-history characteristics, and vagility, we intend to resolve intraspecific spatial genetic patterns representative of a range of elasmobranchs in this region. We discuss the implications of our findings in light of fisheries management and conservation in the Arabian Peninsula.

## Materials and Methods

### Sample collection and DNA extraction

Tissue samples of *C. sorrah* and *R. acutus* were collected between 2010 and 2013 from whole sharks at fish markets and landing sites in Saudi Arabia (Red Sea coast), Oman, the United Arab Emirates (UAE), and Bahrain; *C. limbatus* and *S. lewini* were collected from all locations except Bahrain (site 17, Fig.1), where these species were uncommon or absent in a previous landings survey (Moore and Peirce 2013). Details of species-specific sample numbers per landing site are given in Table S1.

Animals were initially identified based on morphological characteristics. Saudi Arabian samples were obtained from one fish market only (Jeddah), but landings at this site originated from fishing grounds spanning the country's entire Red Sea coast (Spaet and Berumen 2015) (Fig.1). Samples from the UAE were collected from landing and market sites in Abu Dhabi, Dubai, Sharjah, and Ras Al Khaimah as described in Jabado et al. (2014a, 2015). Samples from Oman were collected directly from landing sites along the Omani coast; samples from Bahrain were obtained at the wholesale market of the capital, Manama (Fig.1; Table S1).

At all collection sites, special care was taken to avoid inclusion of specimens for which catch location data were unavailable. This was achieved by interviewing fishermen and traders onsite and verifying the obtained information by a thorough assessment of license plates and origin information of transport trucks used. Based on interpreted assisted fishermen interviews, in the Red Sea, 90% of all four species originated from the five main landing sites displayed in Fig.1, from where they were transported to the Jeddah market by trucks. The remaining 10% originated from smaller landing sites along the Saudi Arabian Red Sea coast. Based on the limited operating range of fishing vessels in Saudi Arabia, all fishing grounds were assumed to lie within a 1–30 km radius of the landing sites. While hence no exact catch location data were available, all samples from Jeddah could definitely be assigned to the Red Sea Basin. The operational range of vessels landing into sites in Oman tends to be small, generally limited to within a few kilometers of the landing site (Henderson et al. 2007). Fishermen in the UAE remain in Gulf waters, yet they are known to travel up to 130–185 km from their landing sites to find productive fishing grounds (Jabado et al. 2014b). The majority of Bahrain specimens were caught in local Bahraini waters (Moore and Peirce 2013) although some may have come from nearby Saudi Arabian or Qatari waters. Despite extensive efforts to determine exact catch locations for more detailed seascape genetic analyses, it was not always possible to assign the origin of samples to their respective landing site regions with 100% certainty. As a precautionary approach, all genetic analyses were hence run with pooled data for the two main geographic groups, combining all samples obtained from the Red Sea into one group (Red Sea) and all samples obtained from outside the Red Sea into a second group representing other Arabian basins (OAB), that is, the Arabian Sea, the Gulf of Oman, and the Gulf (Fig.1).

At all market locations, small fin clips or gill tissue were collected from each specimen and preserved in 99% ethanol. Total genomic DNA was extracted from 10 to 20 mg of preserved tissue using the Macherey-Nagel Genomic DNA from tissue extraction kit (Bethlehem, PA) following the manufacturer's instructions and subsequently stored at −80°C until further analysis.

### Microsatellites – laboratory methods and data analysis

Shark samples were genotyped at 8 to 12 microsatellite loci (*C. limbatus*, 12 loci; *C. sorrah*, 9 loci; *R. acutus*, 8 loci; *S. lewini*, 12 loci). Microsatellite loci were adopted from Feldheim et al. (2001), Keeney and Heist (2003), Ovenden et al. (2006), and Nance et al. (2009) and were directly applied to target species or cross-amplified in nontarget species. Between two and three multiplex PCRs were performed per individual for all species. PCRs were performed in 11 *μ*L total volume containing 2 *μ*L genomic DNA, 5 *μ*L Qiagen Multiplex PCR Master Mix, 3.5 *μ*L H_2_0, and 0.5 *μ*L of primer mix (each primer at 2 *μ*mol/L). Thermal profiles consisted of a denaturation step at 95°C for 15 min, followed by 30 cycles of 30 sec at 94°C, annealing for 90 sec at loci-specific temperatures between 55°C and 60°C (Table S2), and an extension of 60 sec at 72°C, with a final extension of 30 min at 60°C. Fragment analysis was conducted in an Applied Biosystems 3730 XL genetic analyzer, and microsatellite alleles were scored using GENEMAPPER software (v4.0 Applied Biosystems, Foster City, CA). The null hypothesis of Hardy–Weinberg equilibrium (HWE) was tested using GENEPOP on the Web (v4.2 Rousset 2008). MICRO-CHECKER (v2.2.3 van Oosterhout et al. 2004) was used to determine likely causes for deviations from HWE. GENEPOP was also used to characterize genetic diversity (expected (H_E_), observed (H_O_) and unbiased (UH_E_) heterozygosity, allelic richness, and mean number of alleles.

STRUCTURE (v2.3.4 Pritchard et al. 2000) was used to infer the number of putative discrete populations in all samples. We set *K* = 1–10 for each run, assuming prior population information and an admixture model allowing for mixed ancestry of individuals. Each run was repeated three times with independent allele frequencies, 100,000 steps, and a burn-in of 10,000 steps. We used STRUCTURE Harvester (Earl 2012) to determine which K best describes the data according to the highest averaged maximum-likelihood score and Evanno's delta K (Evanno et al. 2005). We then re-ran STRUCTURE with pooled data for the two main geographic groups, combining all samples obtained from the Red Sea into one group (Red Sea) and all samples obtained from the OABs into a second group (Fig.1). A hierarchical analysis of molecular variance (AMOVA) implemented in ARLEQUIN (v3.5 Excoffier and Lischer 2010) and *F*_ST_ (Weir and Cockerham 1984) values were calculated using ARLEQUIN. All microsatellite *F*_ST_ values were corrected (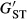 in Hedrick (2005)) using SMOGD (v1.2.5 Crawford 2010) to compensate for the downward bias in *F*_ST_ associated with highly variable microsatellites.

### Mitochondrial DNA – laboratory methods and data analysis

For each species, we examined genetic subdivision based on sequence variation in the mtDNA CR. Approximately 1120 base pairs (bp) of the 5′end of the mtDNA CR was amplified for *C. limbatus*, *C. sorrah,* and *R. acutus* using the forward primer ProL2 and the reverse primer PheCacaH2 (Pardini et al. 2001). A different primer set was used for *S. lewini* to identify potential specimens of the recently described cryptic species *S. gilberti* (Quattro et al. 2013). The forward primer CRF6 and the reverse primer CRR10 (Pinhal et al. 2012) were shown to clearly distinguish between the two species and were hence used in our study to amplify approximately 700 bp of the initial portion of the mtDNA CR for all *S. lewini* specimens. Amplification protocols were the same for both primers and followed those described in Spaet and Berumen (2015). For *S. lewini* and *R. acutus,* 700 bp and 1021 bp of the CR were sequenced in the forward and reverse direction, respectively. For *C. limbatus* and *C. sorrah,* approximately 600 bp of the CR, respectively, was sequenced in the forward direction only, but to ensure accuracy of nucleotide designations, rare and questionable haplotypes were sequenced in both directions. The program Codon Code Aligner (v4.7.2 CodonCode Corporation, Dedham, MA) was used to assemble, check, manually edit, and subsequently align sequences using the MUSCLE algorithm. Aligned sequences were exported to FaBox (Villesen 2007) and collapsed into haplotypes. Initial species identifications based on morphological characters during market sampling were confirmed by comparison with reference CR sequences in the GenBank database through BLAST (http://blast.ncbi.nlm.nih.gov/Blast.cgi). In the case of *R. acutus*, no reference sequences were available prior to this study. Therefore, to validate initial species identification, all *R. acutus* samples were amplified for the COI gene using the primer combination Fish F1 and Fish R1 (Ward et al. 2005). The PCR protocol used was identical to the one used for the CR locus. PCR products were purified and sequenced following Spaet and Berumen (2015). Resultant COI sequences were compared to reference sequences in GenBank (http://www.ncbi.nlm.nih.gov) for species recognition. If sequence data did not match the original identification, respective specimens (*C. limbatus*: three, *C. sorrah*: four, *S. lewini*: eight, *R. acutus*: seven) were excluded from the data set.

For *C. limbatus, C. sorrah*, and *S. lewini*, haplotype networks were constructed to explore the relationships among intraspecific haplotypes. Published haplotypes were sourced from Keeney et al. (2003, 2005), Keeney and Heist (2006), and Sodré et al. (2012) for *C. limbatus*; Giles et al. (2014) for *C. sorrah*; and Duncan et al. (2006), Chapman et al. (2009), and Nance et al. (2011) for *S. lewini*, aligned with novel haplotypes for each species, trimmed to one length (*C. limbatus*: 554 bp, *C. sorrah*: 455 bp, *S. lewini*: 534 bp), and subsequently assessed using a statistical parsimony network constructed in TCS (v1.21 Clement et al. 2000). For *R. acutus*, a parsimony network was constructed based on the haplotypes recorded in this study.

An AMOVA under the Tamura–Nei (TN) model of sequence evolution, which was individually selected as the most appropriate model for all four species in jModelTest (v2.1.4. Darriba et al. 2012), was used to assess population genetic structure in ARLEQUIN. ARLEQUIN was also used to describe the genetic variation between the Red Sea and OAB sampling regions by haplotype and nucleotide diversity (*h* and *π,* respectively).

Ramos-Onsins and Rozas (2002) demonstrated that Fu's *F*_s_ neutrality test (Fu 1997) has the greatest power to detect population expansion for non-recombining regions, such as mtDNA, under a variety of different circumstances, when population sample sizes are large (>50). We hence calculated Fu's *F*_s_ to assess deviations from selective sequence neutrality that could be attributed to selection and/or population size changes. Significance was tested with 100,000 permutations. Recent population expansion is indicated by negative (and significant) *F*_s_ values. The time since the most recent population expansion was estimated by fitting the population parameter *τ* (Rogers and Harpending 1992) for both sampling regions and each species. Mutation rate estimates were available from previous studies for *S. lewini*: 0.8% divergence between lineages per million years or 0.4 × 10^−8^ mutations per site per year (Duncan et al. 2006) and for *C. limbatus*: 0.43% or 0.215 × 10^−8^ (Keeney and Heist 2006); no species-specific mutation rates were available for *C. sorrah* and *R. acutus*. For those species, we hence used the averaged mutation rate (0.62%) reported for other shark species (Galván-Tirado et al. 2013). Generation time estimates were available from previous studies for all four species, *C. limbatus*: 10 years, *C. sorrah*: 4.3 years (Cortés 2002), and *R. acutus*: 2.5 years (Simpfendorfer 2003). Generation time estimates for *S. lewini* are controversial and vary among ocean basins (e.g., Branstetter 1987; Liu and Chen 1999). As no estimates were available for the Indian Ocean, we used the generation time estimated for the closest ocean region for which an estimate was available, the west Pacific: 16.7 years (Cortés 2002). We estimated population expansion times assuming a constant molecular clock and rates using the Mismatch Calculator tool developed by Schenekar and Weiss (2011).

## Results

### Genetic diversity and summary statistics

#### Microsatellites

Microsatellite indices of genetic diversity, that is expected (H_E_), observed (H_O_), and unbiased (UH_E_) heterozygosities, allelic richness, and mean number for each locus and species within each sample region are provided in Table S2. No signs of linkage disequilibrium were detected among any pairs of loci after correction for multiple comparisons.

In all species, several microsatellite loci showed deviations from HWE in one or both of the putative populations and signs of null alleles (Table S2). To test whether significant differences between expected vs. observed heterozygosities at some loci could confound population level analyses, we removed all those loci and re-ran AMOVA analyses. A comparison of *F*_ST_ values calculated from the subset of loci in HWE and from the full data set was not significant for any of the species (paired t-tests calculated in JMP *P *>* *0.6 in all species). To ensure that the pattern of microsatellite structure (or lack thereof) was not being driven by a single locus, we conducted locus-by-locus AMOVA analyses (data not shown), which gave consistent results across all except one locus (Cli118, *C. sorrah*). This locus was solely responsible for the observed pattern of significant population structure and was subsequently removed from the analysis.

#### Mitochondrial DNA

Low haplotype (*h*) and nucleotide (*π*) diversities were found for *C. limbatus* and *S. lewini*, while *C. sorrah* and *R. acutus* showed slightly higher *h* and *π* values (Table1).

**Table 1:** Mitochondrial DNA control region sample size and genetic diversity indices for *Carcharhinus limbatus*, *C. sorrah*, *Rhizoprionodon acutus*, and *Sphyrna lewini* across both sampling regions. Haplotype (*h*) and nucleotide (*π*) diversities, neutrality statistics (Fu's *F*_S_), and estimates of times since last population expansion are shown. Population expansion ranges (below expansion times) are given for 95% confidence intervals of tau

Species	*n* (Red Sea)	*n* (OAB)	Time since expansion Yrs (Red Sea)	Time since expansion Yrs (OAB)	*h *± SD Red Sea	*h *± SD OAB	*π *± SD Red Sea	*π *± SD OAB	Fu's *F*_S_ Red Sea	Fu's *F*_S_ OAB
*C. limbatus*	172	115	182,772 (96,129–273,696)	269,498 (4.2–1,273,612)	0.3490 ± 0.0387	0.3054 ± 0.0525	0.000724 ± 0.00748	0.000755 ± 0.000769	−2.90484	−1.89320
*C. sorrah*	159	216	214,134 (11–656,541)	178,331 (8551–478,324)	0.3270 ± 0.0479	0.4606 ± 0.0412	0.001030 ± 0.000893	0.001314 ± 0.001408	**−7.23390**	**−7.43393**
*R. acutus*	77	217	190,831 (89,096–358,124)	177,561 (48,023–378,345)	0.7365 ± 0.0345	0.6599 ± 0.0306	0.001397 ± 0.000956	0.001265 ± 0.000881	−3.57008	**−12.17461**
*S. lewini*	82	151	151,245 (71,174–249,110)	139,679 (64,724–194,172)	0.4998 ± 0.0318	0.4661 ± 0.0317	0.000086 ± 0.000232	0.000116 ± 0.00027	0.73386	−0.13352

Numbers in bold are significant, *P *<* *0.02 for Fu's *F*_S_ estimates, Fu (1997).

Fu's *F*_s_ statistics were negative for all four species and both sampling regions, yet significant only for *C. sorrah* for both regions (Fu's *F*_s_ Red Sea = −7.23; *P *=* *0.002; Fu's *F*_s_ OAB = −7.43; *P *=* *0.006) and for *R. acutus* for the OAB region only (Fu's *F*_s_ = −12.17; *P *=* *0.01) (Table1). The range of *τ* values yielded estimates of time since last population expansion with very similar expansion time estimates in all four species and both regions (139.679–269.498 years, Table1).

### Genetic structure

The results obtained from all STRUCTURE runs yielded *K* = 1, indicating no differentiation among tentative populations.

*F*_ST_ values were small and nonsignificant for mtDNA analyses in all four species. Very low, yet significant genetic population subdivision was found using microsatellite allele frequencies for *C. limbatus* (0.012; *P *=* *0.00), *R. acutus* (0.002; *P *=* *0.04), and *S. lewini* (0.006; *P* = 0.001) (Table2).

**Table 2:** *F*_ST_ results and associated *P*-values for both regions, characterizing spatial structure with both mtDNA and microsatellites. Standardized *F*_ST_ values (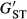, Hedrick 2005) are shown in brackets

	mtDNA	Microsatellites
*Carcharhinus limbatus*	0.0025; *P *=* *0.236	0.012; *P *=* *0.00 (0.0128)
*C. sorrah*	0.0057; *P *=* *0.099	0.000; *P *=* *0.58 (0.000)
*Rhizoprionodon acutus*	0.0608; *P *=* *0.583	0.002; *P *=* *0.04 (0.000864)
*Sphyrna lewini*	0.0130, *P *=* *0.050	0.006; *P *=* *0.001 (0.009604)

#### Mitochondrial DNA

##### Carcharhinus limbatus

**Figure 2:**
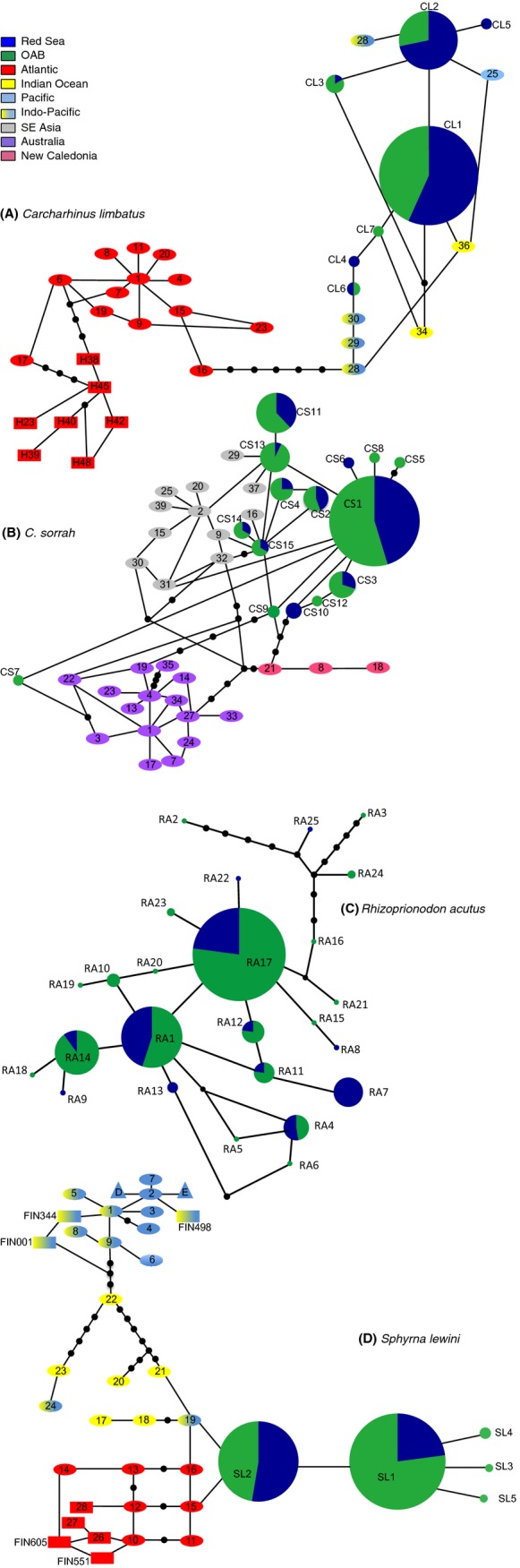
Mitochondrial control region haplotype networks for *Carcharhinus limbatus* (A), *C. sorrah* (B), *Rhizoprionodon acutus* (C), and *Sphyrna lewini* (D) constructed by statistical parsimony in TCS 1.21 (Clement et al. 2000). Circles are sized in proportion to the number of individuals with that haplotype. Each connecting line represents a single mutation. Black dots represent inferred mutational steps. Ocean basins are indicated by colors: The study region is color coded by geographical regions displayed in Fig.1, dark blue (Red Sea), green (OAB). Haplotypes sampled in previous studies are indicated by red (Atlantic), yellow (Indian), light blue (Pacific), yellow fading to blue (shared Indian Pacific), gray (South-East Asia), purple (Australia), salmon (New Caledonia) and are numbered to match their designations in those studies. (A) CL5–CL7 represent novel haplotypes discovered in this study. Haplotypes sampled in previous studies are indicated by ovals (Keeney et al. 2003, 2005; Keeney and Heist 2006) and rectangles (Sodré et al. 2012). CL1–CL4 are identical to Indian Ocean and Indo-Pacific haplotypes discovered by Keeney and Heist (2006). CL1 = H33; CL2 = H24, H26, H27, and H35; CL3 =  H31; and CL4 =  H32. (B) CS4–CS10 and CS12 represent novel haplotypes. Haplotypes sampled by Giles et al. (2014) are represented by ovals. Haplotype CS1 is identical to H5, CS2 to H36, CS3 to H11, CS11 to H12, CS13 to H6, CS14 to H26, and CS15 to H38 in Giles et al. (2014). (D) SL1–SL5 represent novel haplotypes. Haplotypes sampled in previous studies are indicated by ovals (Duncan et al. 2006), rectangles (Chapman et al. 2009), and triangles (Nance et al. 2011).

A 554-bp sequence was obtained for 287 *C. limbatus* individuals. A total of seven haplotypes (GenBank Accession Numbers: KR232982-KR232988) were defined, characterized by five polymorphic sites composed of five transitions (Table S3A). Except for three singletons, all haplotypes were found in both putative populations and matched known Indian Ocean and Indo-Pacific mtCR haplotypes from the global data set of Keeney and Heist (2006). One haplotype (CL1) clearly dominated the sample set and was found in both populations in almost identical numbers (Red Sea: *n* = 100; OAB: *n* = 131). Two singletons were unique to the Red Sea and one was unique to the OAB. Novel haplotypes were very closely related to Indian Ocean and Indo-Pacific haplotypes reported in Keeney and Heist (2006) and at least nine mutational steps away from any Atlantic haplotypes (Fig.2A).

##### Carcharhinus sorrah

A 455-bp sequence was resolved for 375 individuals and resulted in 15 mtDNA haplotypes (GenBank Accession Numbers: KR232989-KR233003), characterized by 12 polymorphic sites composed of 10 transitions, one transversion, and one deletion (Table S3B). All common haplotypes were observed in both putative populations. One haplotype clearly dominated the sample set (CS1). Seven haplotypes matched haplotypes from the range-wide data set of Giles et al. (2014). All novel haplotypes were closely related to Indian Ocean and South-East Asian haplotypes reported in Giles et al. (2014) and formed a lineage distinct from Australian and New Caledonian haplotypes (Fig.2B).

##### Rhizoprionodon acutus

Variation in a 1021-bp fragment of 294 *R. acutus* specimens defined 25 haplotypes (GenBank Accession Numbers: KR232957-KR232981) characterized by 22 polymorphic sites composed of 18 transitions, five transversions, and two deletions (Table S3B). All common haplotypes were separated by two mutational steps at most. Three singletons and one haplotype, recorded from two individuals only, were separated from the cluster of common haplotypes by up to 10 mutational steps. Except for one (RA7) that was unique to the OAB, all common haplotypes were shared in both sampling regions. Haplotype RA17 dominated the sample set and was found in more than half of all OAB samples (Fig.2C).

##### Sphyrna lewini

A 562-bp sequence revealed low levels of diversity for 233 *S. lewini* specimens: five haplotypes (GenBank Accession Numbers: KR232952-KR232956), characterized by four polymorphic sites composed of two transitions and two transversions (Table S3) that differed by no more than one mutational step from each other. Two haplotypes clearly dominated the sample set (Fig2D). All five haplotypes were novel, that is, not present in the global data set of Duncan et al. (2006) or in any of the regional data sets by Chapman et al. (2009), Nance et al. (2011), and Castillo-Olguín et al. (2012). The parsimony network provided evidence that the haplotypes discovered in this study form a Distinct Population Segment (DPS).

## Discussion

This study is the first to assess the population structure of large mobile marine vertebrates between the Red Sea and all other Arabian Ocean Basins. Our analyses were based on a comparatively large number of samples (total *n* = 1189) of four different shark species, from collection locations spanning across over 5000 km of coastline genotyped at two types of genetic markers (mtDNA and nuclear DNA). Contrary to previous findings of significant population genetic structure across the region in different taxa, our results indicate that dispersal of sharks around the Arabian Peninsula is not limited by any obvious barriers to gene flow. Furthermore, ecological, morphological, and life-history differences among the investigated species do not appear to significantly influence their patterns of population structure. Divergent haplotypes in one of our study species (*S. lewini*), however, are suggestive of an Arabian population that is genetically distinct from others in the Indian Ocean.

Several previous studies have shown the existence of historical, oceanographical, and ecological barriers to gene flow resulting in genetic subdivision in a range of marine organisms among ocean basins surrounding the Arabian Peninsula. Our analyses did not provide compelling evidence for more than one Arabian Sea genetic stock for *C. limbatus, C. sorrah, R. acutus*, or *S. lewini*. There was slight evidence of genetic structure between the Red Sea and the OAB for *C. limbatus*, *R. acutus*, and *S. lewini* based on microsatellite allele frequencies; however, *F*_ST_ values were low (0.002–0.012) and not consistent among different statistical tests. These inconsistencies might stem from the high number of null alleles in our data set, which might have been caused by (1) cross-species rather than species-specific loci used in this study due to the limited availability of microsatellite loci for all investigated species and/or (2) species-specific loci, which were developed for specimens sampled in other ocean regions. For future studies on elasmobranch species from regions that have not previously been included in samples used for the design of microsatellite markers, we hence recommend designing species-specific markers based on samples originating from the targeted study region.

The homogenous population structure observed here was not unexpected, given the contiguous shelf habitat around the Arabian Peninsula and the high potential mobility of our study organisms. While previous regional and range-wide studies on *C. limbatus*, *C. sorrah,* and *S. lewini* demonstrated restricted dispersal across deep ocean habitats, genetic structure along continental margins was shown to be relatively minor (Duncan et al. 2006; Keeney and Heist 2006; Nance et al. 2011; Daly-Engel et al. 2012; Giles et al. 2014). Studies on all four species across spatial scales similar to this study in Australia and Indonesia demonstrated heterogeneous population structure in *C. sorrah* and *R. acutus*, but not for *S. lewini* and *C. limbatus* between central Indonesia and northern Australia based on nuclear and mtDNA markers (Ovenden et al. 2009, 2010, 2011). The observed subdivision in the two smaller, less vagile species was suggested to arise from the Timor Trough acting as a deep water dispersal barrier (Ovenden et al. 2009). While large expanses of deep water dividing shallow habitats are absent in our study region, potential oceanographic barriers to gene flow may still exist, for example, regional upwelling systems or local turbid water regions (Schott 1983). Present-day oceanic currents and habitat heterogeneity in the study area have recently been suggested to inhibit gene flow in teleost larval dispersal (DiBattista et al. 2013; Nanninga et al. 2014). Sharks, however, are lacking the dispersive larval phase of most teleost fish, and based on our results, their swimming capacities as juveniles and especially as adults are likely too strong to be influenced by ocean currents characteristic of the Arabian region. Intermittent historical barriers like the ones created by Pleistocene glacial cycles have also reportedly impacted gene flow in teleost species between the Red Sea and the Indian Ocean (Klausewitz 1989; DiBattista et al. 2013). A potential significant reduction in population size during this period was demonstrated by negative and significant indices of neutral evolution (Fu's *F*_s_ test) for *C. sorrah* and *R. acutus,* indicating recent population expansion events between approximately 178,000 and 214,000 years ago (Table1). Those events likely followed substantial bottleneck events that were caused by re-occurring limitations of inflow and exchange of surface water between the Red Sea and the Indian Ocean (Siddall et al. 2003). The decrease in sea water level during those periods likely caused increased evaporation, raising temperatures, and salinity levels beyond the tolerance limits of most marine fauna (Biton et al. 2008). Another reason for the observed excess of low-frequency haplotypes might be caused by positive selection. However, to unambiguously discern between the effects of natural selection and demographic population expansion would necessitate an analysis of several unlinked loci in the genome, because selection only acts on specific loci (Akey et al. 2004).

Additionally to the apparent homogenous population structure, we also found no indication of differences between male and female dispersal in any of the study species. This finding stands in contrast to previous studies describing marked philopatric behavior (Feldheim et al. 2014) in *C. limbatus* and *S. lewini* based on contrasting mitochondrial and nuclear data (Keeney et al. 2003, 2005; Daly-Engel et al. 2012). We suggest that long-shore movements of both males and females along the continuous coastline stretching from the Red Sea all the way into the Gulf cause panmixia over large spatial scales across the region.

Genetic diversity for *C. limbatus* and *S. lewini* was relatively low, with only seven and five haplotypes, respectively. Yet, this pattern appears to be typical for both species throughout their global range (Duncan et al. 2006; Keeney and Heist 2006) and hence may not necessarily be a function of overexploitation. While all our samples of *C. limbatus* and *C. sorrah* matched previously published Indian Ocean and Indo-Pacific mtDNA haplotypes, all haplotypes discovered for *S. lewini* were novel.

There are two possible explanations for the observed genetic separation between *S. lewini* specimens sampled around the Arabian Peninsula and specimens sampled in other, nearby Indian Ocean regions (e.g., the Seychelles and Madagascar, Duncan et al. 2006). First, Arabian Seas *S. lewini* may have evolved to breed differently from conspecifics outside this area. Estuaries have repeatedly been reported as an important nursery habitat for *S. lewini* elsewhere in the world (e.g., Clarke 1971; Snelson 1981; Simpfendorfer and Milward 1993; Duncan and Holland 2006). Due to the desertification of the Arabian region in the past few thousand years, permanent estuaries are now entirely absent for several thousand kilometers of continental coastline from Iraq to Somalia, an area that encompasses our study region. Suggested nursery areas and breeding grounds for *S. lewini*, however, exist near Djibouti City (Bonfil 2003) and in habitats in the central Saudi Arabian Red Sea (J. L. Y. Spaet, unpubl. data), suggesting that the species may not depend on estuarine habitat in these areas. Arabian *S. lewini* may thus have evolved to no longer require estuaries as breeding/nursery grounds, eliminating the need for reproductive migrations and thereby reducing gene flow with other populations. Such scenarios may also explain why *C. limbatus* and *C. sorrah*, which are not reported as being strongly dependent on estuary nurseries, are genetically well connected to other Indian Ocean populations. Second, regional oceanography and upwelling zones may form temporary barriers between Arabian and other Indian Ocean populations. The Somali Current, for instance, which only operates between June and September (Schott 1983), may coincide with key migration/breeding periods of *S. lewini*, but not with those of *C. limbatus* and *C. sorrah*. In this case, mixing with south Indian Ocean populations might be inhibited for *S. lewini,* but not for the other two species. Additional data on migration routes, migration times and breeding cycles, however, are needed to confirm either of these hypotheses.

The fact that Arabian *S. lewini*, which comprise a large amount of the commercial harvest in the Arabian region (Jabado et al. 2015; Spaet and Berumen 2015), might represent a DPS raises a new layer of conservation concern and may warrant species-specific conservation actions under the Convention on International Trade in Endangered Species (CITES), Convention on the Conservation of Migratory Species of Wild Animals (CMS), and a re-evaluation of its IUCN Red List conservation status. Future research should focus on the identification of broader scale genetic breaks by sampling all four species further to the west and east of our sampling locations. In addition, research on dispersal mechanisms based on nongenetic techniques, for example, tagging or parasite studies coupled with molecular methods would provide interesting insights into the actual dispersal mechanisms underlying the observed homogenous population structure.

## Conclusions

Molecular studies on a diverse range of elasmobranch species have done much to illuminate issues that complicate fisheries management and conservation (see Dudgeon et al. 2012 for a review). Here, we provide the first multispecies analysis of population structure between Red Sea and Arabian Sea, Gulf of Oman, and Gulf elasmobranchs indicating that dispersal of four different shark species is not limited by any obvious barriers to gene flow in the waters surrounding the Arabian Peninsula. Three broad conclusions are apparent:

1. Existing contemporary barriers such as regional upwelling systems and ocean currents are likely not influencing long-shore or stepping-stone connectivity even in smaller, less vagile shark species like *R. acutus*.
2. A comparison of novel *S. lewini* haplotypes with published western Indian Ocean haplotypes revealed the possibility of a *S. lewini* population in the Arabian region that is distinct from other Indian Ocean populations.
3. Similar dispersal patterns in sharks with contrasting ecological, morphological, life-history, and distributional patterns indicate that populations of other shark species are likely to also function as common stocks across all ocean basins surrounding the Arabian Peninsula.

Overall, our results call for urgent regional cooperation on the management of shark stocks in all countries surrounding the Arabian Peninsula to ensure a sustainable future for this vital component of the marine biodiversity in the western Indian Ocean. Regulations on the exploitation of only one part of the stock will not suffice and management arrangements need to be implemented, enforced, and coordinated among all responsible authorities. Given current harvesting levels and the apparent connectedness of stocks, unregulated exploitation in one or several countries is likely to cause uniform depletion across the entire stock.

## Data Accessibility

Sequences of all haplotypes presented here have been submitted to GenBank under accession numbers: KR232952-KR233003. In addition the entire data set used in this study has been deposited in the Dryad Data Repository, doi: 10.5061/dryad.4gk47

## Supporting Information

**Table S1**. Number of tissue samples obtained from all landing sites and fish markets for *Carcharhinus limbatus*, *C. sorrah*, *Rhizoprionodon acutus,* and *Sphyrna lewini*. **Table S2.** Microsatellite loci used with their respective annealing temperatures (°C), sample size (*N*), number of alleles (Na), number of effective alleles (Ne), average observed (Ho), expected (He) and unbiased (UHe) heterozygosity, and F statistics for Red Sea and other Arabian basins (OAB), i.e. Arabian Sea, Gulf of Oman and Gulf samples of (A) *Carcharhinus limbatus,* (B) *C. sorrah,* (C) *Rhizoprionodon acutus,* and (D) *Sphyrna lewini*. **Table S3.** Polymorphic nucleotide positions for mitochondrial DNA control region haplotypes for (A) *Carcharhinus limbatus*, (B) *C. sorrah*, (C) *Rhizoprionodon acutus,* and (D) *Sphyrna lewini*. Haplotype numbers, corresponding to Figure 2 are listed in the left columns.

## References

[b1] Ahonen H, Harcourt RG, Stow AJ (2009). Nuclear and mitochondrial DNA reveals isolation of imperilled grey nurse shark populations (*Carcharias taurus*. Mol. Ecol.

[b2] Akey JM, Eberle MA, Rieder MJ, Carlson CS, Shriver MD, Nickerson DA (2004). Population history and natural selection shape patterns of genetic variation in 132 genes. PLoS Biol.

[b3] Andrews KR, Karczmarski L, Au WW, Rickards SH, Vanderlip CA, Bowen BW (2010). Rolling stones and stable homes: social structure, habitat diversity and population genetics of the Hawaiian spinner dolphin (*Stenella longirostris*. Mol. Ecol.

[b4] Baranes A, Randall JE (1989). *Narcine bentuviai*, a new torpedinoid ray from the northern Red Sea. Isr. J. Zool.

[b5] Baum J, Clarke S, Domingo A, Ducrocq M, Lamónaca AF, Gaibar N (2007). http://www.iucnredlist.org.

[b6] Benavides MT, Horn RL, Feldheim KA, Shivji MS, Clarke SC, Wintner S (2011). Global phylogeography of the dusky shark *Carcharhinus obscurus*: implications for fisheries management and monitoring the shark fin trade. Endanger. Species Res.

[b7] Biton E, Gildor H, Peltier WR (2008). Red Sea during the Last Glacial Maximum: implications for sea level reconstruction. Paleoceanography.

[b8] Blower DC, Pandolfi JM, Bruce BD, Gomez-Cabrera MC, Ovenden JR (2012). Population genetics of Australian white sharks reveals fine-scale spatial structure, transoceanic dispersal events and low effective population sizes. Mar. Ecol. Prog. Ser.

[b9] Bonfil R (2003).

[b10] Branstetter S (1987). Age, growth and reproductive biology of the silky shark, *Carcharhinus falciformis*, and the scalloped hammerhead, *Sphyrna lewini*, from the northwestern Gulf of Mexico. Environ. Biol. Fishes.

[b11] Carrier JC, Musick JA, Heithaus MR (2012). Biology of sharks and their relatives.

[b12] Castillo-Olguín E, Uribe-Alcocer M, Díaz-Jaimes P (2012). Assessment of the population genetic structure of *Sphyrna lewini* to identify conservation units in the Mexican Pacific. Cienc. Mar.

[b13] Castro ALF, Stewart BS, Wilson SG, Hueter RE, Meekan MG, Motta PJ (2007). Population genetic structure of Earth's largest fish, the whale shark (*Rhincodon typus*. Mol. Ecol.

[b14] Chapman DD, Pinhal D, Shivji MS (2009). Tracking the fin trade: genetic stock identification in western Atlantic scalloped hammerhead sharks *Sphyrna lewini*. Endanger. Species Res.

[b15] Clarke TA (1971). The ecology of the scalloped hammerhead shark, *Sphyrna lewini*. Hawaii Pacific Sci.

[b16] Clarke CR, Karl SA, Horn RL, Bernard AM, Lea JS, Hazin FH (2015). Global mitochondrial DNA phylogeography and population structure of the silky shark, *Carcharhinus falciformis*. Mar. Biol.

[b17] Clement M, Posada D, Crandall KA (2000). TCS: a computer program to estimate gene genealogies. Mol. Ecol.

[b18] Compagno LJV (2001). Sharks of the world: an annotated and illustrated catalogue of shark species known to date.

[b19] Compagno LJV (1984). FAO species catalogue. Vol. 4. Sharks of the world. An annotated and illustrated catalogue of shark species known to date. Part 2. Carcharhiniformes.

[b20] Cortés E (2002). Incorporating uncertainty into demographic modeling: application to shark populations and their conservation. Conserv. Biol.

[b21] Crawford NG (2010). SMOGD: software for the measurement of genetic diversity. Mol. Ecol. Resour.

[b22] Daly-Engel TS, Seraphin KD, Holland KN, Coffey JP, Nance HA, Toonen RJ (2012). Global phylogeography with mixed-marker analysis reveals male-mediated dispersal in the endangered scalloped hammerhead shark (*Sphyrna lewini*. PLoS ONE.

[b23] Dammannagoda ST, Hurwood DA, Mather PB (2008). Evidence for fine geographical scale heterogeneity in gene frequencies in yellowfin tuna (*Thunnus albacares*) from the north Indian Ocean around Sri Lanka. Fish. Res.

[b24] Darriba D, Taboada GL, Doallo R, Posada D (2012). jModelTest 2: more models, new heuristics and parallel computing. Nat. Methods.

[b25] DiBattista JD, Rocha LA, Craig MT, Feldheim KA, Bowen BW (2012). Phylogeography of two closely related Indo-Pacific butterflyfishes reveals divergent evolutionary histories and discordant results from mtDNA and microsatellites. J. Hered.

[b26] DiBattista JD, Berumen ML, Gaither MR, Rocha LA, Eble JA, Choat JH (2013). After continents divide: comparative phylogeography of reef fishes from the Red Sea and Indian Ocean. J. Biogeogr.

[b27] Dudgeon CL, Broderick D, Ovenden JR (2009). IUCN classification zones concord with, but underestimate, the population genetic structure of the zebra shark *Stegostoma fasciatum* in the Indo-West Pacific. Mol. Ecol.

[b28] Dudgeon CL, Blower DC, Broderick D, Giles JL, Holmes BJ, Kashiwagi T (2012). A review of the application of molecular genetics for fisheries management and conservation of sharks and rays. J. Fish Biol.

[b29] Duncan KM, Holland KN (2006). Habitat use, growth rates and dispersal patterns of juvenile scalloped hammerhead sharks *Sphyrna lewini* in a nursery habitat. Mar. Ecol. Prog. Ser.

[b30] Duncan KM, Martin AP, Bowen BW (2006). Global phylogeography of the scalloped hammerhead shark (*Sphyrna lewini*. Mol. Ecol.

[b31] Earl DA (2012). STRUCTURE HARVESTER: A website and program for visualizing STRUCTURE output and implementing the Evanno method. Conserv. Genet. Resour.

[b32] Evanno G, Regnaut S, Goudet J (2005). Detecting the number of clusters of individuals using the software STRUCTURE: a simulation study. Mol. Ecol.

[b33] Excoffier L, Lischer HEL (2010). Arlequin suite ver 3.5: a new series of programs to perform population genetics analyses under Linux and Windows. Mol. Ecol. Resour.

[b34] Feldheim KA, Gruber SH, Ashley MV (2001). Population genetic structure of the lemon shark (*Negaprion brevirostris*) in the western Atlantic: DNA microsatellite variation. Mol. Ecol.

[b35] Feldheim KA, Gruber SH, DiBattista JD (2014). Two decades of genetic profiling yields first evidence of natal philopatry and long-term fidelity to parturition sites in sharks. Mol. Ecol.

[b36] Foote AD, Vilstrup JT, De Stephanis R (2011). Genetic differentiation among North Atlantic killer whale populations. Mol. Ecol.

[b37] Fratini S, Vannini M (2002). Genetic differentiation in the mud crab *Scylla serrata* (Decapoda: Portunidae) within the Indian Ocean. J. Exp. Mar. Biol. Ecol.

[b39] Fu Y-X (1997). Statistical tests of neutrality of mutations against population growth, hitchhiking and background selection. Genetics.

[b40] Galván-Tirado C, Díaz-Jaimes P, García-de León FJ, Galván-Magaña F, Uribe-Alcocer M (2013). Historical demography and genetic differentiation inferred from the mitochondrial DNA of the silky shark (*Carcharhinus falciformis*) in the Pacific Ocean. Fish. Res.

[b41] Giles JL, Ovenden JR, Al Mojil D, Garvilles E, Khampetch K-o, Manjebrayakath H (2014). Extensive genetic population structure in the Indo-West Pacific spot-tail shark, *Carcharhinus sorrah*. Bull. Mar. Sci.

[b42] Giles EC, Saenz-Agudelo P, Hussey NE, Ravasi T, Berumen ML Exploring seascape genetics and kinship in the reef sponge *Stylissa carteri* in the Red Sea. Ecol. Evol.

[b43] Hedrick PW (2005). A standardized genetic differentiation measure. Evolution.

[b44] Henderson AC, McIlwain JL, Al Oufi HS, Ambu-Ali A (2006). Reproductive biology of the milk shark *Rhizoprionodon acutus* and the bigeye houndshark *Iago omanensis* in the coastal waters of Oman. J. Fish Biol.

[b45] Henderson AC, McIlwain JL, Al-Oufi HS, Al-Sheili S (2007). The Sultanate of Oman shark fishery: species composition, seasonality and diversity. Fish. Res.

[b46] Henderson AC, McIlwain JL, Al-Oufi HS, Al-Sheile S, Al-Abri N (2009). Size distributions and sex ratios of sharks caught by Oman's artisanal fishery. Afr. J. Mar. Sci.

[b47] Hoelzel AR, Shivji MS, Magnussen J, Francis MP (2006). Low worldwide genetic diversity in the basking shark (*Cetorhinus maximus*. Biol. Lett.

[b48] Hoolihan JP, Anandh P, van Herwerden L (2006). Mitochondrial DNA analyses of narrow-barred Spanish mackerel (*Scomberomorus commerson*) suggest a single genetic stock in the ROPME sea area (Arabian Gulf, Gulf of Oman, and Arabian Sea). ICES J. Mar. Sci.

[b49] Hoolihan JP, Premanandh J, D'Aloia-Palmier M-A (2004). Intraspecific phylogeographic isolation of Arabian Gulf sailfish *Istiophorus platypterus* inferred from mitochondrial DNA. Mar. Biol.

[b50] Jabado RW, Al Ghais SM, Hamza W, Henderson AC (2014b). The shark fishery in the United Arab Emirates: an interview based approach to assess the status of sharks. Aquat. Conserv.

[b51] Jabado RW, Al Ghais SM, Hamza W, Henderson AC, Shivji MS (2014a). Shark diversity in the Arabian/Persian Gulf higher than previously thought: insights based on species composition of shark landings in the United Arab Emirates. Mar. Biodivers.

[b52] Jabado RW, Al Ghais SM, Hamza W, Henderson AC, Spaet JLY, Shivji MS (2015). The trade in sharks and their products in the United Arab Emirates. Biol. Conserv.

[b53] Karl SA, Castro ALF, Garla RC (2012). Population genetics of the nurse shark (*Ginglymostoma cirratum*) in the western Atlantic. Mar. Biol.

[b54] Karl SA, Castro ALF, Lopez JA, Charvet P, Burgess GH (2011). Phylogeography and conservation of the bull shark (*Carcharhinus leucas*) inferred from mitochondrial and microsatellite DNA. Conserv. Genet.

[b55] Keeney DB, Heist EJ (2003). Characterization of microsatellite loci isolated from the blacktip shark and their utility in requiem and hammerhead sharks. Mol. Ecol. Notes.

[b56] Keeney DB, Heist EJ (2006). Worldwide phylogeography of the blacktip shark (*Carcharhinus limbatus*) inferred from mitochondrial DNA reveals isolation of western Atlantic populations coupled with recent Pacific dispersal. Mol. Ecol.

[b57] Keeney DB, Heupel M, Hueter RE, Heist EJ (2003). Genetic heterogeneity among blacktip shark, *Carcharhinus limbatus*, continental nurseries along the US Atlantic and Gulf of Mexico. Mar. Biol.

[b58] Keeney DB, Heupel M, Hueter RE, Heist EJ (2005). Microsatellite and mitochondrial DNA analyses of the genetic structure of blacktip shark (*Carcharhinus limbatus*) nurseries in the northwestern Atlantic, Gulf of Mexico, and Caribbean Sea. Mol. Ecol.

[b59] Klausewitz W (1989). Evolutionary history and zoogeography of the Red Sea ichthyofauna. Fauna of Saudi Arabia.

[b60] Kochzius M, Blohm D (2005). Genetic population structure of the lionfish *Pterois miles* (Scorpaenidae, Pteroinae) in the Gulf of Aqaba and northern Red Sea. Gene.

[b61] Kohler NE, Turner PA (2001). Shark tagging: a review of conventional methods and studies. Environ. Biol. Fishes.

[b62] Kohler NE, Casey JG, Turner PA (1998). NMFS Cooperative Shark Tagging Program, 1962-93: an atlas of shark tag and recapture data. Mar. Fish. Rev.

[b63] Kunal SP, Kumar G, Menezes MR, Meena RM (2013). Mitochondrial DNA analysis reveals three stocks of yellowfin tuna *Thunnus albacares* (Bonnaterre, 1788) in Indian waters. Conserv. Genet.

[b64] Last PR, Matsumoto BMM, Moore A (2012). *Himantura randalli* sp. nov., a new whipray (Myliobatoidea: Dasyatidae) from the Persian Gulf. Zootaxa.

[b65] Lewallen EA, Anderson TW, Bohonak AJ (2007). Genetic structure of leopard shark (*Triakis semifasciata*) populations in California waters. Mar. Biol.

[b66] Liu K-M, Chen C-T (1999). Demographic analysis of the scalloped hammerhead shark, *Sphyrna lewini*, in the Northwestern Pacific. Fish. Sci.

[b67] Möller L, Valdez FP, Allen S, Bilgmann K, Corrigan S, Beheregaray LB (2011). Fine-scale genetic structure in short-beaked common dolphins (*Delphinus delphis*) along the East Australian Current. Mar. Biol.

[b68] Moore ABM (2011). Elasmobranchs of the Persian (Arabian) Gulf: ecology, human aspects and research priorities for their improved management. Rev. Fish Biol. Fisheries.

[b69] Moore ABM, White WT, Ward RD, Naylor GJP, Peirce R (2011). Rediscovery and redescription of the smoothtooth blacktip shark, *Carcharhinus leiodon* (Carcharhinidae), from Kuwait, with notes on its possible conservation status. Mar. Freshw. Res.

[b70] Moore ABM, Peirce R (2013). Composition of elasmobranch landings in Bahrain. Afr. J. Mar. Sci.

[b71] Moore ABM, McCarthy ID, Carvalho GR, Peirce R (2012). Species, size, sex and male maturity composition of previously unreported elasmobranch landings in Kuwait, Qatar and Abu Dhabi Emirate. J. Fish Biol.

[b72] Nance HA, Daly-Engel TS, Marko PB (2009). New microsatellite loci for the endangered scalloped hammerhead shark, *Sphyrna lewini*. Mol. Ecol. Resour.

[b73] Nance HA, Klimley P, Galván-Magaña F, Martínez-Ortíz J, Marko PB (2011). Demographic processes underlying subtle patterns of population structure in the scalloped hammerhead shark, *Sphyrna lewini*. PLoS ONE.

[b74] Nanninga GB, Saenz-Agudelo P, Manica A, Berumen ML (2014). Environmental gradients predict the genetic population structure of a coral reef fish in the Red Sea. Mol. Ecol.

[b75] Naylor GJP, Caira JN, Jensen K, Rosana KAM, White WT, Last PR (2012). A DNA sequence-based approach to the identification of shark and ray species and its implications for global elasmobranch diversity and parasitology. Bull. Am. Mus. Nat. Hist.

[b76] Ovenden JR, Street R, Broderick D (2006). New microsatellite loci for Carcharhinid sharks (*Carcharhinus tilstoni* and *C**sorrah*) and their cross-amplification in other shark species. Mol. Ecol. Notes.

[b77] Ovenden JR, Kashiwagi T, Broderick D, Salini J (2009). The extent of population genetic subdivision differs among four co-distributed shark species in the Indo-Australian archipelago. BMC Evol. Biol.

[b78] Ovenden JR, Morgan JAT, Kashiwagi T, Broderick D, Salini J (2010). Towards better management of Australia's shark fishery: genetic analyses reveal unexpected ratios of cryptic blacktip species *Carcharhinus tilstoni* and *C**limbatus*. Mar. Freshw. Res.

[b79] Ovenden JR, Morgan JAT, Street R, Tobin A, Simpfendorfer C, Macbeth W (2011). Negligible evidence for regional genetic population structure for two shark species *Rhizoprionodon acutus* (Rüppell, 1837) and *Sphyrna lewini* (Griffith, Smith, 1834) with contrasting biology. Mar. Biol.

[b80] Pardini AT, Jones CS, Noble LR, Kreiser B, Malcolm H, Bruce BD (2001). Sex-biased dispersal of great white sharks. Nature.

[b81] Pilcher NJ, Antonopolou M, Perry L, Abdel-Moati MA, Al Abdessalaam TZ, Albeldawi M (2014). Identification of Important Sea Turtle Areas (ITAs) for hawksbill turtles in the Arabian Region. J. Exp. Mar. Biol. Ecol.

[b82] Pinhal D, Shivji MS, Vallinoto M, Chapman DD, Gadig OBF, Martins C (2012). Cryptic hammerhead shark lineage occurrence in the western South Atlantic revealed by DNA analysis. Mar. Biol.

[b83] Pomilla C, Amaral AR, Collins T, Minton G, Findlay K, Leslie MS (2014). The World's Most Isolated and Distinct Whale Population? Humpback Whales of the Arabian Sea. PLoS ONE.

[b84] Portnoy DS, McDowell JR, Heist EJ, Musick JA, Graves JE (2010). World phylogeography and male-mediated gene flow in the sandbar shark, *Carcharhinus plumbeus*. Mol. Ecol.

[b85] Pritchard JK, Stephens M, Donnelly P (2000). Inference of population structure using multilocus genotype data. Genetics.

[b86] Quattro JM, Driggers WB, Grady JM, Ulrich GF, Roberts MA (2013). *Sphyrna gilberti* sp nov, a new hammerhead shark (Carcharhiniformes, Sphyrnidae) from the western Atlantic Ocean. Zootaxa.

[b87] Ramos-Onsins SE, Rozas J (2002). Statistical properties of new neutrality tests against population growth. Mol. Biol. Evol.

[b88] Rogers AR, Harpending H (1992). Population growth makes waves in the distribution of pairwise genetic differences. Mol. Biol. Evol.

[b89] Rousset F (2008). GENEPOP'007: a complete re-implementation of the GENEPOP software for Windows and Linux. Mol. Ecol. Resour.

[b90] Schenekar T, Weiss S (2011). High rate of calculation errors in mismatch distribution analysis results in numerous false inferences of biological importance. Heredity.

[b91] Schmidt JV, Schmidt CL, Ozer F, Ernst RE, Feldheim KA, Ashley MV (2009). Low genetic differentiation across three major ocean populations of the whale shark, *Rhincodon typus*. PLoS ONE.

[b92] Schott F (1983). Monsoon response of the Somali Current and associated upwelling. Prog. Oceanogr.

[b93] Schrey AW, Heist EJ (2003). Microsatellite analysis of population structure in the shortfin mako (*Isurus oxyrinchus*. Can. J. Fish Aquat. Sci.

[b94] Schultz JK, Feldheim KA, Gruber SH, Ashley MV, McGovern TM, Bowen BW (2008). Global phylogeography and seascape genetics of the lemon sharks (genus Negaprion). Mol. Ecol.

[b95] Sheppard CRC, Price ARG, Roberts CM (1992). Marine ecology of the Arabian Region: patterns and processes in extreme tropical environments.

[b96] Shih H-T, Saher NU, Kamrani E, Ng PK, Lai Y-C, Liu M-Y (2015). Population genetics of the fiddler crab *Uca sindensis* (Alcock, 1900)(Crustacea: Brachyura: Ocypodidae) from the Arabian Sea. Zool. Stud.

[b97] Siddall M, Rohling EJ, Almogi-Labin A, Hemleben Ch, Meischner D, Schmelzer I (2003). Sea-level fluctuations during the last glacial cycle. Nature.

[b98] Simpfendorfer CA (2003). http://www.iucnredlist.org.

[b99] Simpfendorfer CA, Milward NE (1993). Utilisation of a tropical bay as a nursery area by sharks of the families Carcharhinidae and Sphyrnidae. Environ. Biol. Fishes.

[b100] Snelson FF (1981). Notes on the occurrence, distribution, and biology of elasmobranch fishes in the Indian River lagoon system, Florida. Estuaries.

[b101] Sodré D, Rodrigues-Filho LF, Souza RF, Rego PS, Schneider H, Sampaio I (2012). Inclusion of South American samples reveals new population structuring of the blacktip shark (*Carcharhinus limbatus*) in the western Atlantic. Genet. Mol. Biol.

[b102] Spaet JLY, Berumen ML (2015). Fish market surveys indicate unsustainable elasmobranch fisheries in the Saudi Arabian Red Sea. Fish. Res.

[b103] Spaet JLY, Cochran JEM, Berumen ML (2011). First record of the Pigeye Shark, *Carcharhinus amboinensis* (Müller & Henle, 1839) (Carcharhiniformes: Carcharhinidae), in the Red Sea. Zool. Middle East.

[b104] Spaet JLY, Thorrold SR, Berumen ML (2012). A review of elasmobranch research in the Red Sea. J. Fish Biol.

[b105] Stevens JD, West GJ, McLoughlin KJ (2000). Movements, recapture patterns, and factors affecting the return rate of carcharhinid and other sharks tagged off northern Australia. Mar. Freshw. Res.

[b106] Toonen RJ, Andrews KR, Baums IB (2011). Defining boundaries for applying ecosystem-based management: a multispecies case study of marine connectivity across the Hawaiian Archipelago. J. Mar. Biol.

[b107] van Oosterhout C, Hutchinson WF, Wills DPM, Shipley P (2004). MICRO-CHECKER: software for identifying and correcting genotyping errors in microsatellite data. Mol. Ecol. Notes.

[b108] Vignaud TM, Maynard JA, Leblois R, Meekan MG, Vázquez-Juárez R, Ramírez D (2014b). Genetic structure of populations of whale sharks among ocean basins and evidence for their historic rise and recent decline. Mol. Ecol.

[b109] Vignaud TM, Mourier J, Maynard JA, Leblois R, Spaet JLY, Clua E (2014a). Blacktip reef sharks, *Carcharhinus melanopterus*, have high genetic structure and varying demographic histories in their Indo-Pacific range. Mol. Ecol.

[b110] Villesen P (2007). FaBox: an online toolbox for fasta sequences. Mol. Ecol. Notes.

[b111] Ward RD, Zemlak TS, Innes BH, Last PR, Hebert PD (2005). DNA barcoding Australia's fish species. Philos. Trans. R. Soc. Lond. B Biol. Sci.

[b112] Weir BS, Cockerham CC (1984). Estimating F-statistics for the analysis of population structure. Evolution.

[b113] Zemlak TS, Ward RD, Connell AD, Holmes BH, Hebert PD (2009). DNA barcoding reveals overlooked marine fishes. Mol. Ecol. Resour.

