# Supplementary material for: Population genetics of four heavily exploited shark species around the Arabian Peninsula
Source: Ecol Evol. 2015 May 20;5(12):2317–32. doi: 10.1002/ece3.1515 (PMC4475365; doi:10.1002/ece3.1515)
Supplement: Supplementary file 1 — Table S1. Number of tissue samples obtained from all landing sites and fish markets for Carcharhinus limbatus, C. sorrah, Rhizoprionodon acutus, and Sphyrna lewini. Table S2. Microsatellite loci used with their respective annealing temperatures (°C), sample size (N), number of alleles (Na), number of effective alleles (Ne), average observed (Ho), expected (He) and unbiased (UHe) heterozygosity, and F statistics for Red Sea and other Arabian basins (OAB), i.e. Arabian Sea, Gulf of Oman and Gulf samples of (A) Carcharhinus limbatus, (B) C. sorrah, (C) Rhizoprionodon acutus, and (D) Sphyrna lewini. Table S3. Polymorphic nucleotide positions for mitochondrial DNA control region haplotypes for (A) Carcharhinus limbatus, (B) C. sorrah, (C) Rhizoprionodon acutus, and (D) Sphyrna lewini. Haplotype numbers, corresponding to Figure 2 are listed in the left columns. [file ece30005-2317-sd1.docx]

**SUPPORTING INFORMATION**

| **Table S1**: Number of tissue samples obtained from all landing sites and fish markets for *Carcharhinus limbatus*, *C. sorrah*, *Rhizoprionodon acutus,* and *Sphyrna lewini*. | | | | | |
| --- | --- | --- | --- | --- | --- |
| Regions | Landing sites | *C. limbatus* | *C. sorrah* | *R. acutus* | *S. lewini* |
| Red Sea | Jeddah (1) | 172 | 159 | 77 | 82 |
| OAB (Arabian Sea, Gulf of Oman, the Gulf) | Salalah (2) | 2 | 13 | 2 | 18 |
|  | Mirbat (3) | 4 | 4 | - | - |
|  | Masirah (4) | 7 | 5 | 54 | 27 |
|  | Sur (5) | - | 7 | 2 | 14 |
|  | Muscat (6) | 6 | 17 | 3 | 28 |
|  | Seeb (7) | 1 | 10 | - | - |
|  | Barka (8) | 2 | 3 | - | - |
|  | Sohar (9) | - | - | - | 10 |
|  | Shinas (10) | - | - | - | 10 |
|  | Dibba (11) | 2 | 8 | 13 | - |
|  | Khasab (12) | 5 | 14 | 17 | 33 |
|  | Ras Al Khaimah (13) | 7 | 33 | 17 | 5 |
|  | Sharjah (14) | 5 | 41 | 49 | 2 |
|  | Dubai (15) | 29 | 2 | 4 | 5 |
|  | Abu Dhabi (16) | 45 | 18 | 17 | 1 |
|  | Bahrain (17) | - | 41 | 39 | - |
|  | Total OAB | 115 | 216 | 217 | 151 |
| **Grant total** | | **287** | **375** | **294** | **233** |

**Table S2** Microsatellite loci used with their respective annealing temperatures (°C), sample size (N), number of alleles (Na), number of effective alleles (Ne), average observed (Ho), expected (He) and unbiased (UHe) heterozygosity, and F statistics for Red Sea and other Arabian basins (OAB), i.e. Arabian Sea, Gulf of Oman and Gulf samples of (A) *Carcharhinus limbatus,* (B) *C. sorrah,* (C) *Rhizoprionodon acutus,* and (D) *Sphyrna lewini*. Significant deviations from Hardy-Weinberg Equilibrium are indicated with an asterisk. Potential presence of null alleles is indicated with †.

(A) *Carcharhinus limbatus*

| **region** | **locus** | **T_a_** | **N** | **Na** | **Ne** | **I** | **Ho** | **He** | **UHe** | **F** |  |
| --- | --- | --- | --- | --- | --- | --- | --- | --- | --- | --- | --- |
| **OAB**  **(Arabian Sea, Gulf of Oman, the Gulf)** | **Cli13^a^** | 57 | 108 | 25 | 14.462 | 2.874 | 0.759 | 0.931 | 0.935 | 0.184 | *† |
|  | **Cli55^a^** | 57 | 97 | 15 | 7.744 | 2.261 | 0.722 | 0.871 | 0.875 | 0.171 | *† |
|  | **Cli100^a^** | 55 | 112 | 8 | 4.359 | 1.631 | 0.732 | 0.771 | 0.774 | 0.050 |  |
|  | **Cli102^a^** | 55 | 113 | 11 | 3.881 | 1.663 | 0.611 | 0.742 | 0.746 | 0.177 | *† |
|  | **Cli103^a^** | 57 | 97 | 6 | 3.156 | 1.420 | 0.701 | 0.683 | 0.687 | -0.026 |  |
|  | **Cli107^a^** | 57 | 104 | 12 | 6.161 | 2.026 | 0.788 | 0.838 | 0.842 | 0.059 |  |
|  | **Cli119^a^** | 57 | 116 | 9 | 4.619 | 1.754 | 0.698 | 0.784 | 0.787 | 0.109 |  |
|  | **Cs08^e^** | 57 | 104 | 20 | 11.744 | 2.639 | 0.721 | 0.915 | 0.919 | 0.212 | *† |
|  | **Cs09^e^** | 57 | 109 | 2 | 1.244 | 0.347 | 0.147 | 0.196 | 0.197 | 0.251 | *† |
|  | **Ct05^b^** | 57 | 102 | 12 | 6.305 | 2.027 | 0.657 | 0.841 | 0.846 | 0.219 | *† |
|  | **Ct06^b^** | 55 | 112 | 12 | 6.274 | 2.016 | 0.768 | 0.841 | 0.844 | 0.087 | *† |
|  | **LS24^c^** | 57 | 111 | 7 | 2.406 | 1.180 | 0.486 | 0.584 | 0.587 | 0.167 | *† |
| **Red Sea** | **Cli13^a^** | 57 | 136 | 29 | 14.767 | 2.932 | 0.596 | 0.932 | 0.936 | 0.361 | *† |
|  | **Cli55^a^** | 57 | 141 | 19 | 8.498 | 2.400 | 0.496 | 0.882 | 0.885 | 0.437 | *† |
|  | **Cli100^a^** | 55 | 160 | 10 | 3.945 | 1.648 | 0.688 | 0.747 | 0.749 | 0.079 | * |
|  | **Cli102^a^** | 57 | 163 | 14 | 5.582 | 2.035 | 0.454 | 0.821 | 0.823 | 0.447 | *† |
|  | **Cli103^a^** | 57 | 179 | 9 | 3.609 | 1.545 | 0.620 | 0.723 | 0.725 | 0.142 | *† |
|  | **Cli107^a^** | 57 | 158 | 14 | 6.422 | 2.065 | 0.804 | 0.844 | 0.847 | 0.048 |  |
|  | **Cli119^a^** | 55 | 168 | 11 | 5.116 | 1.844 | 0.732 | 0.805 | 0.807 | 0.090 | *† |
|  | **Cs08^e^** | 57 | 144 | 26 | 12.465 | 2.751 | 0.646 | 0.920 | 0.923 | 0.298 | *† |
|  | **Cs09^e^** | 57 | 179 | 7 | 1.214 | 0.416 | 0.112 | 0.176 | 0.177 | 0.366 | *† |
|  | **Ct05^b^** | 57 | 145 | 11 | 5.866 | 1.919 | 0.697 | 0.830 | 0.832 | 0.160 | *† |
|  | **Ct06^b^** | 55 | 160 | 12 | 6.229 | 2.023 | 0.713 | 0.839 | 0.842 | 0.151 | *† |
|  | **LS24^c^** | 57 | 172 | 8 | 2.378 | 1.174 | 0.529 | 0.580 | 0.581 | 0.087 | * |

(B) C. *sorrah*

| **region** | **locus** | **T_a_** | **N** | **Na** | **Ne** | **Ho** | **He** | **UHe** | **F** |  |
| --- | --- | --- | --- | --- | --- | --- | --- | --- | --- | --- |
| **OAB** | **Cli12^a^** | 55 | 188 | 13 | 1.393 | 0.271 | 0.282 | 0.283 | 0.038 | * |
|  | **Cli100^a^** | 55 | 197 | 6 | 1.150 | 0.102 | 0.130 | 0.131 | 0.221 | *† |
|  | **Cli107^a^** | 55 | 185 | 14 | 5.814 | 0.730 | 0.828 | 0.830 | 0.119 | *† |
|  | **Ct06^b^** | 55 | 158 | 23 | 12.304 | 0.968 | 0.919 | 0.922 | -0.054 |  |
|  | **Cs01^e^** | 55 | 156 | 37 | 23.277 | 0.949 | 0.957 | 0.960 | 0.009 |  |
|  | **Cs02^e^** | 55 | 169 | 34 | 13.738 | 0.917 | 0.927 | 0.930 | 0.011 |  |
|  | **Cs07^e^** | 55 | 190 | 12 | 2.727 | 0.595 | 0.633 | 0.635 | 0.061 | * |
|  | **LS11^c^** | 55 | 197 | 4 | 2.111 | 0.523 | 0.526 | 0.528 | 0.007 |  |
| **Red Sea** | **Cli12^a^** | 55 | 152 | 10 | 1.482 | 0.289 | 0.325 | 0.326 | 0.109 | * |
|  | **Cli100^a^** | 55 | 161 | 6 | 1.227 | 0.112 | 0.185 | 0.186 | 0.396 | *† |
|  | **Cli107^a^** | 55 | 156 | 12 | 5.979 | 0.679 | 0.833 | 0.835 | 0.184 | *† |
|  | **Ct06^b^** | 55 | 137 | 22 | 13.645 | 0.927 | 0.927 | 0.930 | 0 | * |
|  | **Cs01^e^** | 55 | 138 | 40 | 25.025 | 0.906 | 0.960 | 0.964 | 0.057 | *† |
|  | **Cs02^e^** | 55 | 147 | 32 | 15.191 | 0.891 | 0.934 | 0.937 | 0.046 | *† |
|  | **Cs07^e^** | 55 | 163 | 13 | 2.780 | 0.626 | 0.640 | 0.642 | 0.023 | * |
|  | **LS11^c^** | 55 | 160 | 4 | 2.049 | 0.500 | 0.512 | 0.513 | 0.023 | * |

(C) *Rhizoprionodon acutus*

| **region** | **locus** | **T_a_** | **N** | **Na** | **Ne** | **Ho** | **He** | **UHe** | **F** |  |
| --- | --- | --- | --- | --- | --- | --- | --- | --- | --- | --- |
| **OAB** | **Cli07^a^** | 55 | 206 | 17 | 5.444 | 0.757 | 0.816 | 0.818 | 0.072 | *† |
|  | **Cli100^a^** | 55 | 209 | 9 | 6.259 | 0.780 | 0.840 | 0.842 | 0.072 | *† |
|  | **Cli107^a^** | 55 | 206 | 17 | 5.444 | 0.757 | 0.816 | 0.818 | 0.072 | *† |
|  | **Ct03^b^** | 55 | 221 | 7 | 2.502 | 0.615 | 0.600 | 0.602 | -0.025 |  |
|  | **Ct06^b^** | 55 | 217 | 5 | 1.038 | 0.028 | 0.036 | 0.036 | 0.240 | *† |
|  | **LS11^c^** | 55 | 171 | 6 | 1.080 | 0.053 | 0.074 | 0.074 | 0.290 | *† |
|  | **SLE28^d^** | 55 | 223 | 7 | 4.328 | 0.798 | 0.769 | 0.771 | -0.038 |  |
|  | **SLE071^d^** | 55 | 218 | 8 | 2.223 | 0.578 | 0.550 | 0.551 | -0.051 |  |
| **Red Sea** | **Cli07^a^** | 55 | 87 | 12 | 3.650 | 0.540 | 0.726 | 0.730 | 0.256 | *† |
|  | **Cli100^a^** | 55 | 74 | 11 | 7.126 | 0.905 | 0.860 | 0.866 | -0.053 |  |
|  | **Cli107^a^** | 55 | 75 | 15 | 5.230 | 0.747 | 0.809 | 0.814 | 0.077 |  |
|  | **Ct03^b^** | 55 | 89 | 5 | 2.387 | 0.607 | 0.581 | 0.584 | -0.044 |  |
|  | **Ct06^b^** | 55 | 86 | 5 | 1.140 | 0.093 | 0.123 | 0.123 | 0.241 |  |
|  | **LS11^c^** | 55 | 68 | 4 | 1.061 | 0.015 | 0.058 | 0.058 | 0.745 | *† |
|  | **SLE28^d^** | 55 | 87 | 7 | 4.985 | 0.736 | 0.799 | 0.804 | 0.080 | * |
|  | **SLE071^d^** | 55 | 83 | 8 | 2.545 | 0.518 | 0.607 | 0.611 | 0.147 | *† |

| (D) *Sphyrna lewini* | | | | | | | | | | |
| --- | --- | --- | --- | --- | --- | --- | --- | --- | --- | --- |
| **region** | **locus** | **T_a_** | **N** | **Na** | **Ne** | **Ho** | **He** | **UHe** | **F** |  |
| **OAB** | **SLE013^d^** | 60 | 193 | 27 | 3.998 | 0.497 | 0.750 | 0.752 | 0.337 | *† |
|  | **SLE018^d^** | 60 | 197 | 8 | 2.346 | 0.406 | 0.574 | 0.575 | 0.292 | *† |
|  | **SLE025^d^** | 60 | 199 | 34 | 10.416 | 0.804 | 0.904 | 0.906 | 0.111 | *† |
|  | **SLE027^d^** | 60 | 187 | 12 | 5.089 | 0.829 | 0.803 | 0.806 | -0.032 |  |
|  | **SLE028^d^** | 60 | 202 | 31 | 14.573 | 0.866 | 0.931 | 0.934 | 0.070 | *† |
|  | **SLE033^d^** | 60 | 197 | 14 | 4.240 | 0.807 | 0.764 | 0.766 | -0.056 |  |
|  | **SLE038^d^** | 60 | 190 | 14 | 5.646 | 0.863 | 0.823 | 0.825 | -0.049 |  |
|  | **SLE045^d^** | 60 | 204 | 6 | 2.535 | 0.647 | 0.606 | 0.607 | -0.069 | * |
|  | **SLE053^d^** | 60 | 192 | 21 | 10.329 | 0.849 | 0.903 | 0.906 | 0.060 | *† |
|  | **SLE054^d^** | 60 | 206 | 7 | 3.016 | 0.641 | 0.668 | 0.670 | 0.041 |  |
|  | **SLE086^d^** | 60 | 197 | 8 | 1.833 | 0.437 | 0.454 | 0.456 | 0.039 |  |
|  | **SLE089^d^** | 60 | 192 | 14 | 7.935 | 0.870 | 0.874 | 0.876 | 0.005 |  |
| **Red Sea** | **SLE013^d^** | 60 | 69 | 15 | 5.386 | 0.536 | 0.814 | 0.820 | 0.342 | *† |
|  | **SLE018^d^** | 60 | 82 | 8 | 2.799 | 0.500 | 0.643 | 0.647 | 0.222 | *† |
|  | **SLE025^d^** | 60 | 76 | 24 | 10.502 | 0.882 | 0.905 | 0.911 | 0.026 |  |
|  | **SLE027^d^** | 60 | 76 | 9 | 5.176 | 0.882 | 0.807 | 0.812 | -0.093 |  |
|  | **SLE028^d^** | 60 | 76 | 32 | 16.044 | 0.855 | 0.938 | 0.944 | 0.088 | *† |
|  | **SLE033^d^** | 60 | 82 | 13 | 5.255 | 0.817 | 0.810 | 0.815 | -0.009 |  |
|  | **SLE038^d^** | 60 | 79 | 11 | 5.658 | 0.785 | 0.823 | 0.829 | 0.047 | * |
|  | **SLE045^d^** | 60 | 81 | 6 | 2.646 | 0.704 | 0.622 | 0.626 | -0.131 |  |
|  | **SLE053^d^** | 60 | 74 | 18 | 9.203 | 0.838 | 0.891 | 0.897 | 0.060 | * |
|  | **SLE054^d^** | 60 | 80 | 10 | 2.639 | 0.600 | 0.621 | 0.625 | 0.034 |  |
|  | **SLE086^d^** | 60 | 83 | 11 | 2.181 | 0.530 | 0.542 | 0.545 | 0.021 | * |
|  | **SLE089^d^** | 60 | 80 | 15 | 8.573 | 0.888 | 0.883 | 0.889 | -0.005 |  |

**Table S3** Polymorphic nucleotide positions for mitochondrial DNA control region haplotypes for (A) *Carcharhinus limbatus*, (B) *C. sorrah*, (C) *Rhizoprionodon acutus,* and (D) *Sphyrna lewini*. Haplotype numbers, corresponding to Figure 2 are listed in the left columns. The positions of polymorphic base pairs are listed across the top row. The nucleotide at each position is given for haplotype 1. Only nucleotides different from haplotype 1 are given for all other haplotypes. Nucleotides identical to haplotype 1 are indicated with dashes (-) and deletions are indicated with dots (.). Complete haplotype sequences are deposited in GenBank (Accession numbers: KR232952-KR233003).

| (B) *C. sorrah* (455 bp) | | | | | | | | | | | | |
| --- | --- | --- | --- | --- | --- | --- | --- | --- | --- | --- | --- | --- |
| Haplotype | 5  4 | 1  3  9 | 2  4  9 | 2  7  5 | 2  7  8 | 3  3  1 | 3  3  7 | 3  9  8 | 4  0  9 | 4  1  2 | 4  1  3 | 4  1  4 |
| CS1 | A | T | A | C | A | C | A | T | A | A | T | G |
| CS2 | - | - | - | T | - | T | - | C | - | - | - | - |
| CS3 | - | - | - | T | - | - | - | - | - | - | - | - |
| CS4 | - | - | - | T | - | T | - | - | - | - | - | - |
| CS5 | - | - | G | - | - | - | - | - | - | - | - | - |
| CS6 | - | - | - | - | - | - | - | - | - | G | - | - |
| CS7 | - | - | - | T | - | - | G | - | - | - | - | - |
| CS8 | - | C | - | - | - | - | - | - | - | - | - | - |
| CS9 | - | - | - | - | G | T | - | C | - | - | - | - |
| CS10 | - | - | - | - | - | - | G | - | - | - | - | - |
| CS11 | T | - | - | - | - | T | - | - | - | - | - | - |
| CS12 | - | - | - | - | - | - | - | - | G | - | C | . |
| CS13 | - | - | - | - | - | T | - | - | - | - | - | - |
| CS14 | - | - | - | - | - | - | - | C | - | - | - | - |
| CS15 | - | - | - | - | - | T | - | C | - | - | - | - |
|  |  |  |  |  |  |  |  |  |  |  |  |  |
|  |  |  |  |  |  |  |  |  |  |  |  |  |
|  |  |  |  |  |  |  |  |  |  |  |  |  |
|  |  |  |  |  |  |  |  |  |  |  |  |  |

| (A) *Carcharhinus limbatus* (554 bp) | | | | | |
| --- | --- | --- | --- | --- | --- |
| Haplotype | 8  5 | 2  7  7 | 3  2  1 | 5  4  6 | 5  4  7 |
| CL1 | T | A | G | A | T |
| CL2 | - | - | - | G | - |
| CL3 | - | - | A | G | - |
| CL4 | - | G | - | G | - |
| CL5 | - | - | - | G | C |
| CL6 | C | G | - | G | - |
| CL7 | - | G | - | - | - |

| *Rhizoprionodon acutus* (1021 bp) | | | | | | | | | | | | | | | | | | | | | | |
| --- | --- | --- | --- | --- | --- | --- | --- | --- | --- | --- | --- | --- | --- | --- | --- | --- | --- | --- | --- | --- | --- | --- |
| Haplotype | 1  3  7 | 3  2  6 | 3  3  5 | 3  8  4 | 4  9  6 | 5  0  3 | 5  0  4 | 5  9  3 | 6  0  0 | 6  0  8 | 6  0  9 | 6  1  0 | 6  1  1 | 6  1  4 | 6  7  1 | 6  7  5 | 6  9  2 | 6  9  3 | 7  4  5 | 7  8  4 | 9  0  7 | 9  7  8 |
| RA1 | T | C | A | T | C | A | A | T | A | G | C | A | T | T | A | T | G | T | C | A | T | T |
| RA2 | - | T | - | - | - | - | G | - | G | - | . | - | C | C | - | C | A | C | - | - | - | - |
| RA3 | - | T | - | - | - | G | - | C | G | A | T | C | C | C | - | - | - | - | - | - | - | C |
| RA4 | - | T | T | - | - | - | - | - | - | - | - | - | - | - | - | - | - | - | - | - | - | - |
| RA5 | - | T | G | - | - | - | - | - | - | - | - | - | - | - | - | - | - | - | - | - | - | - |
| RA6 | C | T | T | - | - | - | - | - | - | - | - | - | - | - | - | - | - | - | - | - | - | - |
| RA7 | - | - | - | C | - | - | - | - | - | - | - | - | - | - | - | - | - | - | - | G | - | - |
| RA8 | - | T | - | - | A | - | - | - | - | - | - | - | - | - | - | - | - | - | - | - | - | C |
| RA9 | - | - | - | - | - | - | - | - | - | - | - | - | - | - | - | - | - | C | - | - | C | - |
| RA10 | - | - | - | - | - | - | - | - | - | - | - | - | - | - | - | - | - | - | T | - | - | - |
| RA11 | - | - | - | C | - | - | - | - | - | - | - | - | - | - | - | - | - | - | - | - | - | - |
| RA12 | - | - | - | C | - | - | - | - | - | - | - | - | - | - | - | - | - | - | - | - | - | C |
| RA13 | C | - | - | - | - | - | - | - | - | - | - | - | - | - | - | - | - | - | - | - | - | - |
| RA14 | - | - | - | - | - | - | - | - | - | - | - | - | - | - | - | - | - | C | - | - | - | - |
| RA15 | - | - | - | - | - | - | - | - | - | C | - | - | - | - | - | - | - | - | - | - | - | C |
| RA16 | - | - | - | - | A | - | - | - | - | - | - | - | - | - | - | - | - | - | - | - | - | C |
| RA17 | - | - | - | - | - | - | - | - | - | - | - | - | - | - | - | - | - | - | - | - | C | C |
| RA18 | - | - | - | - | - | - | - | - | - | - | - | - | - | - | - | - | - | - | - | - | - | C |
| RA19 | - | - | - | - | - | - | - | - | - | - | - | - | - | - | T | - | - | C | - | - | - | - |
| RA20 | - | - | - | - | - | - | - | - | - | - | - | - | - | - | - | - | - | - | T | - | - | C |
| RA21 | - | - | - | - | - | - | - | - | - | - | - | - | - | - | - | - | - | - | T | - | C | - |
| RA22 | - | - | - | - | - | - | - | - | - | - | . | - | - | C | - | - | - | - | - | - | - | C |
| RA23 | - | - | - | - | - | - | - | - | - | - | - | . | - | C | - | - | - | - | - | - | - | C |
| RA24 | - | T | - | - | - | - | - | - | G | - | . | - | C | C | - | - | - | - | - | - | - | C |
| RA25 | - | T | - | - | - | G | - | - | G | - | . | - | C | C | - | - | - | - | - | G | - | - |
|  |  |  |  |  |  |  |  |  |  |  |  |  |  |  |  |  |  |  |  |  |  |  |

| (D) *Sphyrna lewini* (535 bp) | | | | |
| --- | --- | --- | --- | --- |
| Haplotype | 1  0  5 | 3  1  0 | 5  3  1 | 5  3  2 |
| SL1 | A | C | G | T |
| SL2 | - | - | - | C |
| SL3 | - | T | - | C |
| SL4 | - | - | C | C |
| SL5 | C | C | - | C |
